# Eristic reasoning: Adaptation to extreme uncertainty

**DOI:** 10.3389/fpsyg.2023.1004031

**Published:** 2023-02-09

**Authors:** Rasim Serdar Kurdoglu, Marc Jekel, Nüfer Yasin Ateş

**Affiliations:** ^1^Faculty of Business Administration, Bilkent University, Ankara, Turkey; ^2^Faculty of Human Sciences, University of Cologne, Cologne, Germany; ^3^Sabancı Business School, Sabancı University, Istanbul, Türkiye

**Keywords:** eristic, biases, hedonic goals, extreme uncertainty, self-serving beliefs

## Abstract

Heuristics (shortcut solution rules) can help adaptation to uncertainty by leading to sufficiently accurate decisions with little information. However, heuristics would fail under extreme uncertainty where information is so scarce that any heuristic would be highly misleading for accuracy-seeking. Thus, under very high levels of uncertainty, decision-makers rely on heuristics to no avail. We posit that eristic reasoning (i.e., self-serving inferences for hedonic pursuits), rather than heuristic reasoning, is adaptive when uncertainty is extreme, as eristic reasoning produces instant hedonic gratifications helpful for coping. Eristic reasoning aims at hedonic gains (e.g., relief from the anxiety of uncertainty) that can be pursued by self-serving inferences. As such, eristic reasoning does not require any information about the environment as it instead gets cues introspectively from bodily signals informing what the organism hedonically needs as shaped by individual differences. We explain how decision-makers can benefit from heuristic vs. eristic reasoning under different levels of uncertainty. As a result, by integrating the outputs of formerly published empirical research and our conceptual discussions pertaining to eristic reasoning, we conceptually criticize the fast-and-frugal heuristics approach, which implies that heuristics are the only means of adapting to uncertainty.

## 1. Introduction

Heuristics are short-cut solution rules that reduce effort and time for decision-making (Kahneman, 2003; Shah and Oppenheimer, 2008). Heuristics can produce biased decisions in the sense that decisions may be inaccurate due to incongruencies with probability theory (e.g., conjunction rule; Ahmad et al., 2020; Kahneman, 2003). However, heuristics can be crucially less susceptible to noise and inaccuracy in an uncertain environment than complicated probabilistic calculations (Artinger et al., 2015; also see Hertwig and Gigerenzer, 1999). As such, the use of heuristics can be ecologically rational as they can lead to sufficiently accurate, quick and effective decisions under uncertainty (Gigerenzer, 2008). Yet, such effective uses of heuristics depend on sensing critical (non-compensatory) heuristic cues (i.e., cues that can be used by a heuristic), which can compensate for the lack of probabilistic information (Baron, 2006). Heuristic reasoning cannot be the only means of adapting to uncertainty, especially when uncertainty levels are so high that one cannot recognize heuristic cues.

The concept of heuristics is becoming like a garbage bin, as anything that cannot be explained by logic and probability is often attributed to heuristics (Shah and Oppenheimer, 2008). It is possible that people do not always adapt to uncertainty by using heuristics (Newell et al., 2003; Navarrete and Santamaría, 2011). Adaptation is likely to be achieved with a wide variety of strategies involving disparate motivations during judgement and decision-making (Tetlock, 2002). In this respect, we identify eristic reasoning as an adaptation strategy that functions differently than that realized by heuristics. In heuristic decision-making, decisions are made to satisfy desires by intelligently processing the cues in the external environment. As such, the use of heuristics represents an intelligent strategy to deal with uncertainty, as heuristics are tools of intendedly rational decision-making (Simon, 1978, 1990). By intended rationality, we refer to the procedural rationality norm of Simon (1978, 1990), who suggests that a decision-maker is intendedly rational when she follows rationality as a process where she strives to make a judgement based on the calculation of decision consequences. In comparison, in eristic decision-making, decisions are made by blindly following desires through self-serving illusory beliefs. Eristic reasoning serves purely hedonic goals that can be achieved without the need to sense the heuristic cues in the environment. For instance, one can decide eristically by superstitions (Morisseau et al., 2021) or by wishful thinking (Bhattacharya et al., 2018).

Particularly under extreme uncertainty, it is more adaptive to change the intention of reasoning and shift from intelligent and intendedly rational methods to irrational methods where the decision is guided by self-serving hedonic inferences that are shaped by individual differences. In this regard, a neurotic person would have different hedonic needs than a dopaminergic person under extreme uncertainty. A neurotic person is overly anxious, pessimistic and unconfident (Sharma et al., 2014). Such a person would hedonically need anxiety-relieving and risk aversion. By contrast, a dopaminergic person is overly unconcerned about the future, optimistic and confident (Daw et al., 2006). The hedonic needs of a dopaminergic person would be sensation-seeking and risk-seeking. Thus, people with different personalities would react to uncertainty in distinct ways because of their distinct hedonic needs. Likewise, people can respond to stress differently in their risk-taking depending on their level of social anxiety (Hengen and Alpers, 2021). Yet, except for studies on the risk-sensitivity theory that accounts for the varying needs of individuals (Mishra, 2014), the psychological interaction between individual differences and the external environment is often neglected in the literature on decision-making under uncertainty. What we suggest is that human action does not always follow the tenets of the computational theory of mind, where decision-making is assumed to be handled almost exclusively by intelligent calculations, as championed by Simon (1983).

Irrational eristic inferences can be purposeful and, therefore, potentially adaptive, as every human action is goal-driven in some way or another (Mises, 1988). We posit that irrationality is adaptive for its eristic nature, i.e., winning-oriented thinking with disrespect for truth. For instance, acting on untruthful or superstitious beliefs can be adaptive: such beliefs can artificially decrease the anxiety that is caused by uncertainty, which in turn can boost ensuing performance (e.g., Damisch et al., 2010; Risen, 2016). Similarly, wishful thinking (e.g., Seybert and Bloomfield, 2009) and non-accuracy-seeking motivated reasoning (e.g., Gershman, 2019) can be adaptive for providing instant hedonic gratification.

While people usually have multiple goals in their minds when making their decisions, they have to prioritize them in their decision (Kung and Scholer, 2021). As an intendedly rational approach, heuristics serve the goals that can be achieved by solving the problem at hand *via* truth-seeking. By contrast, eristically made irrational decisions serve the goals that can be achieved without seeking a truthful solution to the problem at hand. In this respect, people preferring eristic reasoning over heuristic reasoning prioritize immediate hedonic goals such as anxiety-relieving, pleasure-seeking, bonding, sensation-seeking, etc., which are shaped by individual differences. While both heuristic and eristic reasoning can eventually serve hedonic goals, eristic reasoning does not involve the first step of the truth-seeking present in heuristic reasoning. As such, eristic reasoning directly targets instant hedonic gratification as opposed to indirect hedonic gratification that can be attained by first pursuing accuracy in problem-solving. For instance, smoking cigarette is an eristic decision to directly satisfy hedonic urges (albeit in a non-adaptive way). Yet, the harms of smoking would normally deter a person from smoking if one rationally considers the long-term hedonic consequences of smoking.

By introducing a novel conceptual distinction between the eristic nature of irrational reasoning and intendedly rational heuristic reasoning, we assert that some of the eminent biases (i.e., the overconfidence bias, the endowment effect, status quo bias, loss aversion, and wishful thinking) are more attributable to eristic reasoning than heuristic reasoning. This is not just a matter of labelling: We posit that eristic reasoning and related biases are adaptive to extreme uncertainty by providing instant hedonic gratifications useful to cope with the unknown. Accordingly, our main theoretical prediction is that individuals are likely to rely on eristic reasoning rather than heuristic reasoning when the uncertainty level is high to extreme. That is to say, when uncertainty approaches extreme levels, accuracy-seeking becomes so infeasible that one needs to listen to her desires blindly as shaped by their personality traits.

## 2. Eristic vs. heuristic reasoning

The term “eristic” originates from the argumentation literature (Perelman, 1982; Wolf, 2010; Kurdoglu and Ateş, 2022), in which eristic arguments are contrasted with heuristic arguments. The literature suggests that eristic arguments signify reasoning to win a debate with disrespect for truth. Eristic arguments are purely winning-oriented and directly interest-seeking moves. By contrast, heuristic arguments signify reasoning to find a sensible and impartial solution to the problem at hand. For instance, judges are supposed to argue and make their judgements heuristically as a disinterested party, whereas lawyers are predicted to argue eristically to defend the interests of their clients in a one-sided manner. Because individuals mainly use reasoning for arguing (Perelman and Olbrechts-Tyteca, 1969; Mercier and Sperber, 2011; Mercier, 2013), the terms’ heuristic vs. eristic’ can be moved into the realm of individual reasoning and decision-making from the realm of argumentation (Kurdoglu et al., 2022; Kurdoglu and Ateş, 2022).

Eristic reasoning is initiated by myths, passions, prejudices and vested interests (Perelman, 1979, 1982). These factors directly respond to individuals’ psychological or material comfort. Heuristic reasoning is not blind to personal well-being either. However, heuristic reasoning seeks personal well-being indirectly by first aiming at “real” problem-solving that depends on accuracy. Solving problems, in turn, can help to achieve well-being. By contrast, eristic reasoning directly aims at personal well-being by spurious inferences. For instance, myths in the form of superstitions are observed to be helpful for psychological comfort under uncertainty (Tsang, 2011; Hamerman and Morewedge, 2015; Risen, 2016; Walco and Risen, 2017). Myths are unfounded beliefs that are not backed by reliable evidence (such as conspiracy theories or belief in karma), while they can be psychologically comforting or frightening. Similarly, passions deteriorate our capacity to decide impartially regarding what we are passionate about. Passions are strong identity-setting emotional attachments to certain activities, people, objects and ideas (Vallerand et al., 2003). When we are passionate about an activity, idea, person or object, we inherently get pleasure from it (Ho et al., 2011). “Following passions” constitute an eristic shortcut in this respect. Prejudices, such as in-group favoritism and out-group derogation, are almost the opposite of passions in the sense that prejudices create a negative emotional distance to a person, object or idea (Hewstone et al., 2002). Finally, vested interests can cause people to sacrifice seeking accuracy to attain instant hedonic gratification. For instance, if a doctor is afraid of being sued for inaction, she may prescribe drugs without questioning their benefits for a particular case.

In comparison to eristic reasoning, heuristic reasoning is about finding a solution through truth-seeking in an efficient way (Shah and Oppenheimer, 2008). In this regard, fast-and-frugal heuristics advocated by Gigerenzer and his followers (e.g., Gigerenzer and Gaissmaier, 2011; Kruglanski and Gigerenzer, 2011; Gigerenzer et al., 2016), such as recognition, fluency, take-the-best, and tallying heuristics, are obviously practical methods of solving a problem at hand by seeking truth efficiently. As such, these heuristics directly address problem-solving goals such as accuracy of perception or efficiency (Felin et al., 2017; Lieder and Griffiths, 2020).

While heuristics can indirectly help to achieve hedonic goals — after all, solving a problem can also make the decision-maker happy—it is the eristic reasoning that seeks hedonic goals in a direct way without truth-seeking. Unlike intendedly rational heuristics or formally rational logic and probability, eristic reasoning produces pleasurable feelings without pursuing the truth in a calculative manner to resolve the problem at hand. For instance, if a financial analyst is searching for the best stock to invest in a profitable way, the financial analyst can directly aim at hedonic gratifications (e.g., increased sensation or reduced anxiety) by eristically picking a stock of a firm simply because the stock label includes a lucky number. However, a good heuristic solution could eventually make her happy as well. For instance, one could look for a heuristic cue (e.g., past performances of stocks) to solve the investment problem with profitable outcomes, which can eventually make the analyst happy. The issue we would like to highlight is that an eristic solution passes the first step of problem-solving by truth-seeking and directly aims at hedonic gratification.

The use of eristics mainly signifies a change in the goals of reasoning. In comparison to reasoning in heuristics, reasoning in eristics does not engage with external reality, and it does not offer intelligent solutions. By contrast, heuristic reasoning engages with external reality to reach satisficing outcomes, and it involves intelligence in problem-solving (e.g., intelligently ignoring part of available information). Yet, as we outlined above, a change in the reasoning goals implies changes in the prioritization of decision goals.

### 2.1. Eristic biases

Eristic reasoning underly various well-known biases, such as the overconfidence bias, the endowment effect, status quo bias, loss aversion, and wishful thinking. The endowment effect blinds people to their belongings’ real market value as people can be hedonically tied to their property. Similarly, loss aversion bias stems from emotional attachment to one’s possessions (Kahneman et al., 1991). Likewise, overconfidence bias indicates a tendency to hedonically overestimate one’s own skills, intellect and talent (Berthet, 2022). In a similar fashion, status quo bias represents a tendency to stick with the existing state of affairs with a close-minded attitude toward alternatives (Gunaydin et al., 2018). In this regard, these eristic biases are products of self-serving inferences, which are not helpful for seeking the truth for an intendedly rational calculation. Such biases involve self-deception and distorted reasoning motivations which are explicitly visible in wishful thinking.

In contrast to eristic biases, heuristic biases involve disregarding some part of the available information (Gigerenzer, 2008) while the aim is truth-seeking or an associated problem-solving goal (i.e., morality or efficiency). The fast-and-frugal heuristics advocated by Gigerenzer and his followers (e.g., Gigerenzer and Gaissmaier, 2011) involve that kind of bias. By contrast, the biases mentioned in the preceding paragraph (i.e., the overconfidence bias, the endowment effect, status quo bias, loss aversion, and wishful thinking), which have been studied by the heuristics-and-biases tradition proponents (e.g., Kahneman, 2003) have a different character that cannot be associated with heuristics by definition. They should be instead associated with eristic reasoning, as explained in the preceding paragraph. However, another set of biases associated with the heuristics-and-biases tradition, namely, representativeness (using similarities to estimate probabilities), availability (focusing on easily recallable memories to make judgements), and anchoring (using a benchmark to make predictions) are still examples of heuristic biases as they aim at accurate decision-making, albeit with imprecision, while saving time and effort (Shah and Oppenheimer, 2008). As such, we believe that our distinction between eristic and heuristic biases can be helpful to alleviate the theoretical dispute between the heuristics-and-biases approach and the fast-and-frugal heuristics approach as both approaches paint the heuristic reasoning with a broad-brush conflating heuristic reasoning with eristic reasoning, therefore producing a confusing theoretical debate.

### 2.2. Abductive calculations of heuristic reasoning vs. self-serving inferences of eristic reasoning

Contrary to the formal rationality of logic and probability, heuristics operate by intendedly rational abductive calculations. Abduction involves using deductive and inductive reasoning iteratively to produce the subjectively most convincing explanation from the available data in a pragmatic fashion (Martela, 2015). As inferences in abductive reasoning do not depend on mathematical or statistical calculations, abductive reasoning makes calculations in a subjective and imperfect way (Behfar and Okhuysen, 2018). By contrast, logic and probability do not allow abductive calculations as they rely on objective calculations driven by deductively built inferences. During the process of abductive reasoning, individuals infer conclusions from their personal knowledge base to make sense of the data they observe (Peirce, 1997). This is consistent with heuristic decision-making processes, in which the drawn knowledge base can be intuitions as well as inductively built experiential and personally or culturally learned knowledge (Denison and Xu, 2019). In comparison to inductive reasoning, in which generalized conclusions are produced from a series of observations, abductive reasoning can produce conclusions even from one observation by heuristically applying prior knowledge to a new situation (Behfar and Okhuysen, 2018).

In comparison to abductive calculations present in heuristic reasoning, eristic inferences operate in a serving inferencing manner to satisfy hedonic goals as shaped by individual differences. The sources of self-serving inferences are the hedonic needs of the individual rather than the heuristic cues present in the environment. Rather than relying on abductive calculations present in heuristic reasoning, eristic reasoning relies on directionally motivated cognition directed to satisfy hedonic urges (e.g., Hughes and Zaki, 2015). While heuristic cues are processed for an intendedly rational abductive search for truth, such as for purposes of foraging (Bella-Fernández et al., 2021), hedonic needs are processed to form self-serving eristic inferences. Overall, rather than responding to goals associated with accuracy-seeking and problem-solving, eristic reasoning responds to hedonic needs, which can be satisfied in a self-serving manner. Yet, eristic reasoning is not a foolish move as it can be adaptive under extreme uncertainty.

## 3. Extreme uncertainty and adaptiveness of eristic reasoning

We define extremely uncertain environments based on three criteria: (1) Environments that are subjectively new and thus have not yet been experienced or explored by the decision-maker in the past, (2) environments in which not just probabilistic quantitative information seems to be lacking but also qualitative information seems to be scarce for the decision-maker after a thorough information search, and (3) environments in which heuristic cues are either lacking at all or are very weak, and ultimately potentially unreliable as they are untested before in a similar environment. By heuristic cues, we mean cues that are helpful for seeking truth and solving the problem at hand accordingly. For instance, for recruitment decisions in a foreign country, the educational background of candidates constitutes a heuristic cue. Similarly, in medical decisions, symptoms are often primary heuristic cues for diagnosis. In cases of extreme uncertainty, however, heuristic cues can be absent. When heuristic cues are present, they are very ambiguous under extreme uncertainty. For instance, medical doctors may struggle to make a diagnosis after observing the symptoms. Extreme uncertainty can also emerge because of the volatility of the situation (e.g., stock market shocks, war-time conditions), rendering past experiences irrelevant. Similarly, it can happen because of the unprecedented nature of the situation (e.g., pandemic), causing many unknowns. In such circumstances, extreme uncertainty can be resolved if people can seek more information, ask around or familiarize themselves with the issue. Yet, this may not always be possible or affordable during decision-making. As such, people may have to decide without an opportunity to wait for a reduction in the uncertainty levels.

The judgement and decision-making research neglects extremely uncertain environments, despite the fact that they can be related to substantial decisions, while the intendedly rational methods are unfeasible in such environments. For example, a patient may decide on a treatment whose risks are completely unknown (cf., Gigerenzer et al., 2016). Without reliable heuristic cues, a decision for such treatment depends on the feeling of desperation and personality traits rather than an elusive realistic assessment of the treatment. Similarly, decisions about career changes and even long-term mating decisions can also be subject to extreme future uncertainties. Individual differences can precipitate different hedonic goals, such as sensation-seeking or anxiety-relieving. To satisfy such hedonic goals, people can sometimes change their careers without much experience and reliable information about the new job and its future prospects. Likewise, people can choose their partners impulsively. Moreover, entrepreneurs and innovators may sometimes decide to invest in extremely uncertain endeavors because of their impulses stemming from their dopaminergic personalities (Nicolaou et al., 2021). Eristic reasoning is particularly likely to play some role in entrepreneurial decisions under extreme uncertainty, as entrepreneurs often make their investment decisions by following their entrepreneurial passions (Cardon et al., 2009; Mueller et al., 2017; Croce et al., 2020).

Although we do not face highly uncertain decision environments daily, they present frequently enough to be of interest. Moreover, their impact can be substantial for the individuals involved. Since heuristics would be ineffective in forming predictions about outcome performance in such environments, people may need to resort to eristic reasoning to cope with the situation, as will be explained next. In this regard, illuminating what people *maximize beyond outcome performance* helps to better understand adaptive decision-making in those situations.

### 3.1. Eristic reasoning is adaptive under extreme uncertainty

We posit that while heuristic reasoning enables adaptation to uncertainty when uncertainty is at moderate levels, eristic reasoning is instead adaptive under extreme uncertainty. According to the fast-and-frugal heuristics approach, the bias that comes with ignoring some relevant variables by heuristics can be advantageous under uncertainty because an alternative probabilistic or mathematical model comprised of many variables can be more fallible (Gigerenzer and Brighton, 2009). Gigerenzer (2008) presents the situation succinctly: When all relevant parameters are added to a decision-making model, predictions can be highly inaccurate under uncertainty because of noise (increased variance when more variables are added to the model). Hence, it is possible to improve decision-making accuracy by being biased in the selection of parameters for the prediction model, as that would reduce the noise. This is called a variance minimization strategy. The idea is that total accuracy errors essentially stem from prediction biases and aggregate variance (Total Error = prediction biases + Aggregate Variance + Unexplained Error). When fewer parameters are added to the prediction model under uncertainty, predictions would be biased (i.e., prediction biases will be high), but the total error would still decrease as the aggregate variance (i.e., aggregation of variance per each variable) will decline sharply because of having fewer parameters to vary (Gigerenzer and Gaissmaier, 2011).

The variance minimization strategy, which justifies the biases of heuristic rules, omits the potential effects of extreme uncertainty. High levels of uncertainty would extremely raise the effect of heuristic biases and make the prediction errors enormously large in comparison to any possible decrease in total variance gained by focusing on a few deciding factors. As such, under extreme uncertainty, truth-seeking will be so elusive that it would be ecologically more adaptive to abandon truth-seeking completely and focus on anxiety reduction or similar hedonic interests by pursuing eristic reasoning. Therefore, we suggest that people can change their goals depending on the level of uncertainty they face and the utilities they drive from each goal. Accordingly, we posit that people can disengage from accuracy-related goals and switch to other goals when they particularly face higher levels of uncertainty. After such a switch, the solutions offered by eristic reasoning do not make sense for the original goal associated with truth-seeking, whereas it makes sense for the new hedonic goal. In this sense, objectives beyond decision accuracy (such as an eristic method’s potential for emotion regulation and stress management) might be relevant for decision-makers in such environments. Accordingly, we argue that the by-products of the *motivational processes* have been neglected in the heuristic decision-making literature (e.g., Gigerenzer and Gaissmaier, 2011), which focuses almost exclusively on the *outcome* of a decision.

A person’s eristic method is not intendedly rational as it involves reasoning aiming at the satisfaction of emotional urges rather than calculations of consequences of an action. We also recognize that eristic reasoning is irrational from the perspective of theoretical rationality norms (cf., Audi, 2004), as eristic reasoning is not interested in the truthful representation of reality. Yet, eristic reasoning can be instrumentally justifiable as it leads to, for example, a reduction of anxiety and a reduction of stress in the decision process (e.g., Hengen and Alpers, 2021). As such, while eristic reasoning is not intendedly rational as it does not involve calculative reasoning, we recognize that eristic reasoning can be rational from the point of view of instrumental rationality (cf. Domeier et al., 2018) in the sense that eristic reasoning can be instrumental for hedonic aims. Yet, even hedonic gains such as emotional relief can be more appropriately pursued by solving the problem at hand realistically through intendedly rational methods rather than by producing self-serving conclusions through eristic reasoning. Therefore, only under extreme uncertainty does eristic reasoning becomes the adaptive method by changing the intentions since extreme uncertainty precludes intentions of rationality. In comparison, under moderate uncertainty, heuristic reasoning and its intended rationality offer the most adaptive route as consequences can be calculated heuristically, thanks to the existence of reliable cues to assess future consequences.

On the other hand, we do not suggest that eristic reasoning and its biases (e.g., loss aversion, status quo bias, endowment effect) occur only under extreme uncertainty. Rather, we suggest eristic reasoning is particularly adaptive under extreme uncertainty. As such, when extreme uncertainty is identified, it is possible to predict the use of eristics as an adaptation strategy, while eristic reasoning can also be used maladaptively in different circumstances. For instance, many entrepreneurial decisions are marked by extreme uncertainty (Huang and Pearce, 2015; Packard et al., 2017; Foss, 2020). In such circumstances, eristic reasoning that draws on entrepreneurial passion can be responsible for entrepreneurial decisions (de Mol et al., 2020; Lex et al., 2022) rather than predictions made by heuristics. While following passion does not guarantee entrepreneurial success, it is justified in terms of adaptation when there is extreme uncertainty and a need to satisfy hedonic urges. As very high levels of entrepreneurial failure attest (Hogarth and Karelaia, 2011), many entrepreneurial endeavours are likely to be initiated by eristic reasoning driven by passion and other hedonic factors rather than by truth-seeking heuristics involving calculations of consequences. From an adaptation perspective, however, eristic submission to passion is adaptive if the decision suffers from extreme uncertainty. Poor consequences would not change the adaptive properties of eristic reasoning as its adaptiveness does not involve a calculation of consequences anyway.

### 3.2. Adaptive utility function

While Gigerenzer and his colleagues (Gigerenzer, 2008, 2018; Gigerenzer and Gaissmaier, 2011; Artinger et al., 2015) extensively explore prediction accuracy advantages of heuristics under uncertainty, they neglect adaptation through eristic strategies aiming at hedonic goals. We would like to make our point by focusing on the adaptiveness of anxiety relief as a hedonic goal. Assume that an individual faces a problem whose resolution is important for the individual. When uncertainty is zero or low, there would be no anxiety owing to uncertainty. In such a situation, it would be adaptive to exploit intendedly rational methods (logic, probability and heuristics) rather than pursuing hedonic goals through eristic reasoning. Assuming that the applicable formally rational methods (analytical methods that depend on logic and probability) are not too time-consuming or unaffordable, the decision-maker would not resort to heuristic reasoning. As such, the adaptive method of decision-making under negligible uncertainty would be analytical methods rather than heuristic methods. On the other hand, when there is a considerable level of uncertainty, heuristics can outperform logical and probabilistic calculations (Gigerenzer and Brighton, 2009; Gigerenzer and Gaissmaier, 2011). The reason is that heuristics depend on a single or a few decision-making variables (cues), thus becoming less vulnerable to variance relative to logical and probabilistic calculations that take many variables into consideration. The elegance of heuristic decision-making is that salient cues are intuitively recognized by the decision-maker (Filevich et al., 2017). However, under extreme uncertainty, heuristics would be as error-prone as random guesses since the salient cues are not perceived by the decision-maker. Under extreme levels of uncertainty, heuristics become useless as predictions by heuristics would be very misleading. Indeed, pursuing truth in any form of rationality, in general, becomes meaningless, while pursuing hedonic gains through eristic reasoning might provide opportunities for exploration, opening up possibilities of serendipitous outcomes and learning, or at least reduced suffering from extreme uncertainty.

To demonstrate the adaptiveness of eristic reasoning, we propose an “adaptive utility” function comprised of the summation of two elements: Gains from problem-solving by accuracy seeking (gains from intended rationality) and hedonic gains from satisfying emotional urges such as anxiety relief in the face of uncertainty. Both types of gains change as a function of uncertainty and the chosen decision-making method. Gains from intended rationality decrease when the uncertainty level is decreased. By contrast, gains from eristically satisfying hedonic urges increase when the uncertainty level is increased. The adaptive utility function can be presented as shown below.


f(x)=A(x)+M(x)

where,


x:Level of Uncertainty

$$f\left(x\right):\mathrm{Adaptive utility}$$

A(x): Gains from problem-solving by accuracy-seeking (intended rationality gains).

M(x): Gains from eristically satisfying hedonic urges (e.g., anxiety relief).

**Figure 1:**
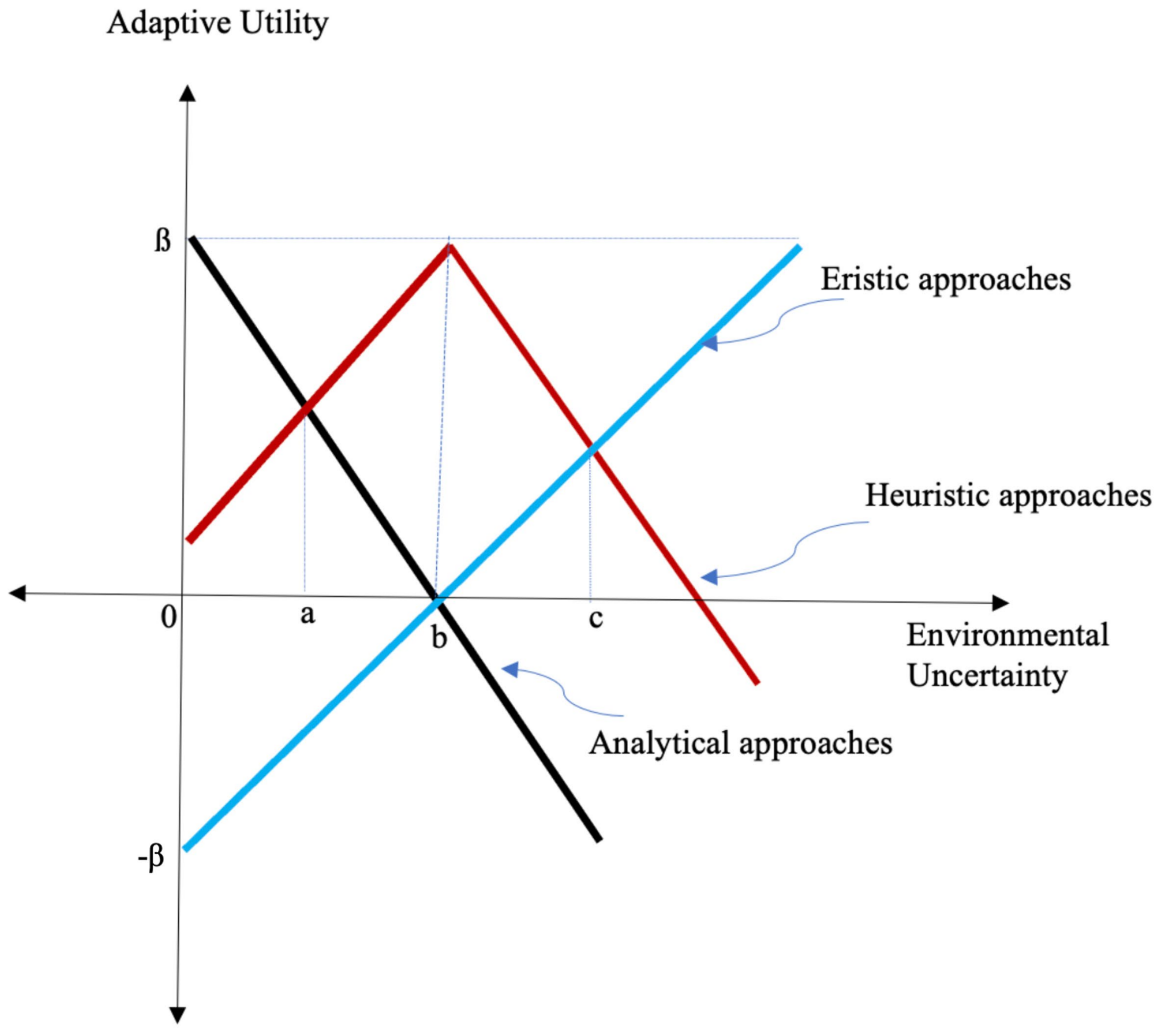
Adaptive utility of decision-making methods under varying uncertainty.

Figure 1 demonstrates how adaptive utility changes for each decision-making approach (analytical methods, heuristic methods and eristic methods) under different levels of uncertainty. In Figure 1, the x-axis represents the level of environmental uncertainty, while the y-axis represents the level of adaptive utility. For simplicity, linear relationships are assumed between uncertainty and adaptive utility for three different decision-making approaches. We assume that the maximum adaptive utility, as well as maximum loss out of any decision-making method, is ß in Figure 1. Per each decision-making approach, there are varying adaptive utility values between ß and-ß. We assume that decision-makers aim at satisficing levels of adaptive utility.

Until the uncertainty level at point a (negligible uncertainty), analytical approaches yield the most adaptive choices as accuracy is easily attainable under low levels of uncertainty. In comparison, at an uncertainty level between point *a* and point *b*, heuristic methods are the ecologically adaptive option as the accuracy of heuristics now tends to be better than analytical approaches. Between point *b* and point *c*, heuristic methods continue to be ecologically more adaptive than eristic methods, but at point c, the adaptive utility of eristic and heuristic methods are equalized as uncertainty levels are getting high. Between points *b* and *c*, eristic methods become increasingly more adaptive for their hedonic gains, while analytical as well as heuristic methods become increasingly less adaptive for their reduced accuracy. This is because, at that interval of uncertainty, the gains attainable from hedonic pursuits (i.e., anxiety relief for this example) are increasing while the gains attainable by pursuing truth are decreasing, although heuristic methods can still outperform eristic methods in terms of producing larger adaptive utility as heuristics methods are still capable of sufficiently accurate predictions. However, further to point c, uncertainty becomes so extreme that it is adaptively more beneficial to pursue decision-making by eristic methods rather than elusively pursuing truth either by heuristic or analytical methods.

### 3.3. An example from game theory

As Shafir and Tversky’s (1992) experiments of the (single-shot) prisoner’s dilemma game demonstrated, individuals indeed decide eristically under uncertainty. When playing the prisoner’s dilemma game, players can either compete (confess the crime) or cooperate (not confess the crime) with each other under the uncertainty of what the other player will do. (i) If both cooperate, they get a short sentence, (ii) if one competes and the other cooperates, only the cooperating one gets a long sentence, (iii) if both compete, both get a medium sentence. From the logical perspective, self-interested utility-maximizing players in the prisoner dilemma game should both compete due to comparative trade-offs to do so under the uncertainty of the other player’s action. This holds true despite the fact that both players would be both better off if they both cooperated. Yet, as Shafir and Tversky’s experiment reported, 37% of 444 participants cooperated against these expectations.

Shafir and Tversky (1992) interpreted the situation as wishful thinking (thinking that the other participant will cooperate as well) or as non-consequentialist evaluation (principled adherence to certain actions) by participants. We agree with their interpretation, but our framework provides a richer explanation: Some individuals engage in wishful thinking or give up consideration of the outcomes for adapting to perceived extreme uncertainty, as their eristic reasoning provides a hedonic relief. As we mentioned, eristic reasoning directly aims at hedonic satisfaction, whereas heuristic reasoning provides hedonic satisfaction *via* problem-solving. Participants who decided to compete might have only perceived moderate uncertainty as they might have presumed that the other party was likely to compete, so they would become vulnerable to exploitation if they cooperated instead. Hence, such a problem-solving approach could be relieving for those participants who perceive moderate uncertainty. By contrast, the participants who decided to cooperate might have thought that the situation was unpredictable, where there was no clue about whether the other party would cooperate or compete. These participants who perceived extreme uncertainty might have resorted to eristic reasoning as a matter of blindly following their desire for the better outcome (i.e., both players are cooperating) by wishfully thinking that the other player can cooperate just like them. It is in this way those participants might have eristically chosen to cooperate to adapt to the extreme uncertainty they perceived.

Our interpretation depends on the condition that under the certainty of the other player’s move, eristic reasoning cannot be the adaptive option as there would be no need for a direct route to hedonic satisfaction. Indeed, a simple modification in the experimental study supports our conclusion. In the same study, Shafir and Tversky conducted the same experiment with a little change: uncertainty of the other player’s action was removed in two different scenarios. In the first scenario, participants were informed that the other player had competed. As expected, 97% of the participant chose to compete in response to the “compete” decision of the other player. In the second scenario, participants were informed that the other player was cooperating. This time, 84% of the participants acted by competing and therefore did not reciprocate the cooperation. Only 16% of the participants cooperated as an ethical reciprocation when the other player was known to be also cooperating. However, the 16% cooperation rate is much lower than the 37% cooperation rate in the original experimental scenario, where there is instead uncertainty about whether the other player is cooperating or not. In other words, when uncertainty is removed in the modified experiment, the participants’ cooperation rate unexpectedly declines. This is puzzling as one would normally expect to see increased cooperation rates once the other party is known to be cooperating as well. It seems to us that when uncertainty is removed in the modified experiment, individuals did not feel the need to directly pursue hedonic relief as they did not perceive extreme uncertainty. In that sense, they instead mostly focused on gains from accurate problem-solving. This can explain why participants became unexpectedly less cooperative when uncertainty was removed.

Similar to the situations portrayed in the prisoner’s dilemma game, people face uncertainties in their relationships with others. We posit that just like people can use heuristic reasoning (e.g., tit for tat, using familiarity to choose mates) to rationally manage some of their interactions with other people (cf. Hertwig et al., 2013), they can also use eristic reasoning to irrationally manage their social relationships with side-taking. Such eristic reasoning can be particularly observed in the reasoning of football fanatics, partisan groups, and religious zealots who are moved by a variety of hedonic drives (e.g., Jost et al., 2003; Kruglanski et al., 2021).

## 4. Some ideas for future research

The distinctions between heuristic and eristic reasoning offer exciting opportunities for future research. First and foremost, future research can study how people are inclined to shift from using heuristics to eristic methods when environmental uncertainty increases. Second, research can identify which eristic methods are preferred under particular scenarios. Third, the roles of eristic reasoning in different domains of decision-making can be explored. For instance, exploring the role of eristic reasoning in moral and political decision-making can be a possible direction for future research. Eristic reasoning can be favorable for political purposes because of its self-serving interest-seeking nature. Yet from a normative perspective, eristic reasoning is not appropriate for principled decision-making that most moral philosophies seek in one way or another. In this respect, while adaptive in some circumstances, eristic methods can nevertheless lead to unethical consequences. As such, further research on eristics can be insightful for studying ethically sensitive issues in different conditions.

As an example of ethical problems, Gigerenzer (2015) mentions how doctors can prescribe unnecessary drugs out of fear of persecution. We believe that such ethically controversial actions are products of the eristic reasoning of doctors who are normally expected to prescribe what is best for the patient. Research can establish antecedents of such eristic moves and thereby identify potential interventions for reducing the application of eristics. Likewise, research on eristic reasoning can shed new light on biases leading to discrimination or misconduct in different contexts, such as hiring at the workplace. As a case of demonstration, we suggest that police misconduct is also possibly related to eristic reasoning. For instance, in shooter-bias experiments (e.g., Johnson et al., 2018), researchers typically present some criminal scenarios to participants where group-based (e.g., racial) stereotypes are the only available distinctive cue for a participant’s decision to shoot or not. In those experiments, racial stereotypes are not heuristically reliable in deciding on using deadly force. Thus, in the absence of any heuristic cue (such as the criminal history of a suspect), an extremely uncertain situation presents itself to participants. In such a situation, the mind may adaptively, though ethically controversially, think eristically and act by following the only available distinctive cue (e.g., race prejudices) that triggers self-serving conclusions. The good news is that in the presence of meaningful heuristic cues, most police officers are unlikely to reason eristically and act solely on their prejudices (Cesario, 2021). In all respects, research can be useful to understand the antecedents and consequences of such ethically controversial uses of eristic reasoning.

At the moment, eristic reasoning can be distinguished from heuristic reasoning by checking for some unique nonlogical elements (i.e., captivating emotions, myths, unfounded prejudices, and vested interests in the reasoning) because these elements have nothing to do with truth-seeking reasoning that is useful for intendedly rational calculations. However, since people may either refuse to accept their true reasoning motivations or they may be unconscious of them, we believe neuroscience methods can be perhaps useful in identifying eristic strategies in decision-making (cf. Volk and Köhler, 2012; Serra, 2021). For instance, as a theoretical possibility, fMRI technology can be utilized to study the changes in the brain’s reward activity during the use of eristic reasoning vs. heuristic reasoning. In particular, through research designs that incorporate economic decision-making games, brain imagining techniques may identify different brain regions that can be associated with eristic and heuristic strategies (Sanfey et al., 2006). As such, it is theoretically possible to discover the neural basis of eristic reasoning. In this respect, neuroscience methods can be perhaps useful to have a definitive biological distinction between heuristic and eristic reasoning. This can be an exciting avenue to explore, particularly for researchers of neuroeconomics (cf. Camerer et al., 2005; Loewenstein et al., 2008; Kable, 2011).

## 5. Conclusion

In this paper, we explicate a useful distinction for the psychological literature on adaptive decision-making as we outline how eristic reasoning is an adaptive alternative to heuristic reasoning under extreme uncertainty (Kurdoglu et al., 2022, 2023). We argue that heuristic methods are, by definition, intendedly rational, whereas eristic methods are not intendedly rational as they are employed to target hedonic goals with self-serving inferences. Overall, we outline how to distinguish heuristic methods from eristic methods, as well as how to distinguish their adaptiveness under varying uncertainty levels. In this respect, we posit that the adaptiveness of decision-making methods should be judged by their intentions at the moment of decision-making and how these intentions match different levels of uncertainty. Under extreme uncertainties, eristics can be adaptive because rationality intentions could be futile under extreme uncertainty while acting eristically would be more adaptive for achieving hedonic gains precipitated by personality characteristics.

Our view enables us to identify an adaptive utility function where we have introduced a new component (satisfaction of hedonic urges) to recast the ecological rationality framework of Gigerenzer and his colleagues (e.g., Gigerenzer and Gaissmaier, 2011). Our adaptation function demonstrates that extreme environmental uncertainties can justify eristically made decisions. For instance, eristic decisions can be adaptive when there is a need to suppress the fear of death and avoid depression (Vail et al., 2012), such as by self-deception, in the face of extreme uncertainties (Perry-Smith and Mannucci, 2017), as was the case during the initial stages of the Covid-19 epidemic (Eden et al., 2020). Yet, when the level of uncertainty is not that high, it is more adaptive to adopt heuristic methods. Further research can empirically test our view by checking whether individuals indeed adjust their decision-making by shifting from using heuristic methods to eristic methods depending on the level of uncertainty they face. We believe studying eristic methods offers an exciting path for future research on adaptive decision-making.

## Author contributions

RK, MJ, and NA contributed to the conception of the study and revised the manuscript. RK wrote the first draft of the manuscript. All authors contributed to the article and approved the submitted version.

## Funding

We acknowledge support for the Article Processing Charge from the DFG (German Research Foundation, 491454339), Bilkent University and Sabancı University. Nüfer Y. Ateş is supported by the Science Academy Young Scientists Award Program (BAGEP) of the Science Academy Society of Turkey.

## Conflict of interest

The authors declare that the research was conducted in the absence of any commercial or financial relationships that could be construed as a potential conflict of interest.

## Publisher’s note

All claims expressed in this article are solely those of the authors and do not necessarily represent those of their affiliated organizations, or those of the publisher, the editors and the reviewers. Any product that may be evaluated in this article, or claim that may be made by its manufacturer, is not guaranteed or endorsed by the publisher.

## References

[ref1] Ahmad M. Shah S. Z. A. Abbass Y. (2020). The role of heuristic-driven biases in entrepreneurial strategic decision-making: evidence from an emerging economy. Manag. Decis. 59, 669–691. doi: 10.1108/MD-09-2019-1231/FULL/PDF

[ref2] Artinger F. Petersen M. Gigerenzer G. Weibler J. (2015). Heuristics as adaptive decision strategies in management. J. Organ. Behav. 36, S33–S52. doi: 10.1002/job.1950

[ref3] Audi R. (2004). “Theoretical rationality: its sources, structure, and scope” in The Oxford handbook of rationality. eds. Mele A. R. Rawling P. (Oxford: Oxford University Press), 17–34.

[ref4] Baron R. A. (2006). Opportunity recognition as pattern recognition: how entrepreneurs ‘connect the dots’ to identify new business opportunities. Acad. Manag. Perspect. 20, 104–119. doi: 10.5465/amp.2006.19873412

[ref5] Behfar K. Okhuysen G. A. (2018). Perspective—discovery within validation logic: deliberately surfacing, complementing, and substituting abductive reasoning in hypothetico-deductive inquiry. Organ. Sci. 29, 323–340. doi: 10.1287/orsc.2017.1193

[ref6] Bella-Fernández M. Suero Suñé M. Gil-Gómez de Liaño B. (2021). Foraging behavior in visual search: a review of theoretical and mathematical models in humans and animals. Psychol. Res. 1, 1–19. doi: 10.1007/S00426-021-01499-1 33745028

[ref7] Berthet V. (2022). The impact of cognitive biases on professionals’ decision-making: a review of four occupational areas. Front. Psychol. 12:802439. doi: 10.3389/FPSYG.2021.802439 35058862 PMC8763848

[ref8] Bhattacharya U. Kuo W. Y. Lin T. C. Zhao J. (2018). Do superstitious traders lose money? Manag. Sci. 64, 3772–3791. doi: 10.1287/mnsc.2016.2701

[ref9] Camerer C. Loewenstein G. Prelec D. (2005). Neuroeconomics: how neuroscience can inform economics. J. Econ. Lit. 43, 9–64. doi: 10.1257/0022051053737843

[ref10] Cardon M. S. Wincent J. Singh J. Drnovsek M. (2009). The nature and experience of entrepreneurial passion. Acad. Manag. Rev. 34, 511–532. doi: 10.5465/amr.2009.40633190

[ref11] Cesario J. (2021). What can experimental studies of bias tell us about real-world group disparities? Behav. Brain Sci. 45:e66. doi: 10.1017/S0140525X21000017, PMID: 33413703

[ref12] Croce A. Ughetto E. Cowling M. (2020). Investment motivations and UK business angels’ appetite for risk taking: the moderating role of experience. Br. J. Manag. 31, 728–751. doi: 10.1111/1467-8551.12380

[ref13] Damisch L. Stoberock B. Mussweiler T. (2010). Keep your fingers crossed! How superstition improves performance. Psychol. Sci. 21, 1014–1020. doi: 10.1177/0956797610372631, PMID: 20511389

[ref14] Daw N. D. O’Doherty J. P. Dayan P. Seymour B. Dolan R. J. (2006). Cortical substrates for exploratory decisions in humans. Nature 441, 441, 876–879. doi: 10.1038/nature04766, PMID: 16778890 PMC2635947

[ref15] de Mol E. Cardon M. S. de Jong B. Khapova S. N. Elfring T. (2020). Entrepreneurial passion diversity in new venture teams: an empirical examination of short-and long-term performance implications. J. Bus. Ventur. 35:105965. doi: 10.1016/j.jbusvent.2019.105965

[ref16] Denison S. Xu F. (2019). Infant statisticians: the origins of reasoning under uncertainty. Perspect. Psychol. Sci. 14, 499–509. doi: 10.1177/1745691619847201 31185184

[ref17] Domeier M. Sachse P. Schäfer B. (2018). Motivational reasons for biased decisions: the sunk-cost effect’s instrumental rationality. Front. Psychol. 9:815. doi: 10.3389/FPSYG.2018.00815 29881366 PMC5976877

[ref18] Eden A. L. Johnson B. K. Reinecke L. Grady S. M. (2020). Media for Coping during COVID-19 social distancing: stress, anxiety, and psychological well-being. Front. Psychol. 11:577639. doi: 10.3389/FPSYG.2020.577639 33391094 PMC7775316

[ref19] Felin T. Koenderink J. Krueger J. I. (2017). Rationality, perception, and the all-seeing eye. Psychon. Bull. Rev. 24, 24, 1040–1059. doi: 10.3758/s13423-016-1198-z, PMID: 27928763 PMC5570804

[ref20] Filevich E. Horn S. S. Kühn S. (2017). Within-person adaptivity in frugal judgments from memory. Psychol. Res. 83, 613–630. doi: 10.1007/S00426-017-0962-7 29273969 PMC6441105

[ref21] Foss N. J. (2020). Behavioral strategy and the COVID-19 disruption. J. Manag. 46, 1322–1329. doi: 10.1177/0149206320945015

[ref22] Gershman S. J. (2019). How to never be wrong. Psychon. Bull. Rev. 26, 26, 13–28. doi: 10.3758/s13423-018-1488-8 29799092

[ref23] Gigerenzer G. (2008). Why heuristics work. Perspect. Psychol. Sci. 3, 20–29. doi: 10.1111/j.1745-6916.2008.00058.x 26158666

[ref24] Gigerenzer G (2015). Simply rational: decision making in the real world. Oxford: Oxford University Press.

[ref25] Gigerenzer G. (2018). The bias bias in behavioral economics. Rev. Behav. Econ. 5, 303–336. doi: 10.1561/105.00000092

[ref26] Gigerenzer G. Brighton H. (2009). Homo heuristicus: why biased minds make better inferences. Top. Cogn. Sci. 1, 107–143. doi: 10.1111/j.1756-8765.2008.01006.x, PMID: 25164802

[ref27] Gigerenzer G. Gaissmaier W. (2011). Heuristic decision making. Annu. Rev. Psychol. 62, 451–482. doi: 10.1146/annurev-psych-120709-145346 21126183

[ref28] Gigerenzer G. Hertwig R. Pachur T. (2016). Heuristics: The foundations of adaptive behavior. Oxford: Oxford University Press.

[ref29] Gunaydin G. Selcuk E. Yilmaz C. Hazan C. (2018). I have, therefore I love: status quo preference in mate choice. Personal. Soc. Psychol. Bull. 44, 589–600. doi: 10.1177/0146167217746339 29276858

[ref30] Hamerman E. J. Morewedge C. K. (2015). Reliance on luck: identifying which achievement goals elicit superstitious behavior. Personal. Soc. Psychol. Bull. 41, 323–335. doi: 10.1177/0146167214565055, PMID: 25617118

[ref31] Hengen K. M. Alpers G. W. (2021). Stress makes the difference: social stress and social anxiety in decision-making under uncertainty. Front. Psychol. 12:578293. doi: 10.3389/FPSYG.2021.578293 33692716 PMC7937725

[ref32] Hertwig R. Gigerenzer G. (1999). The ‘conjunction fallacy’ revisited: how intelligent inferences look like reasoning errors. J. Behav. Decis. Mak. 12 John Wiley and Sons Ltd, 275–305. doi: 10.1002/(SICI)1099-0771(199912)12:4<275::AID-BDM323>3.0.CO;2-M

[ref33] Hertwig R. Hoffrage U. ABC Research Group (2013). Simple Heuristics in a Social World. New York, NY: Oxford University Press.

[ref34] Hewstone M. Rubin M. Willis H. (2002). Intergroup bias. Annu. Rev. Psychol. 53, 575–604. doi: 10.1146/annurev.psych.53.100901.135109 11752497

[ref35] Ho V. T. Wong S.-S. Lee C. H. (2011). A tale of passion: linking job passion and cognitive engagement to employee work performance. J. Manag. Stud. 48, 26–47. doi: 10.1111/J.1467-6486.2009.00878.X

[ref36] Hogarth R. M. Karelaia N. (2011). Entrepreneurial success and failure: confidence and fallible judgment. Organ. Sci. 23, 1733–1747. doi: 10.1287/orsc.1110.0702

[ref37] Huang L. Pearce J. L. (2015). Managing the unknowable: the effectiveness of early-stage investor gut feel in entrepreneurial investment decisions. Adm. Sci. Q. 60, 634–670. doi: 10.1177/0001839215597270

[ref38] Hughes B. L. Zaki J. (2015). The neuroscience of motivated cognition. Trends Cogn. Sci. 19, 62–64. doi: 10.1016/j.tics.2014.12.006 25640642

[ref39] Johnson D. J. Cesario J. Pleskac T. J. (2018). How prior information and police experience impact decisions to shoot. J. Pers. Soc. Psychol. 115, 601–623. doi: 10.1037/pspa0000130 30221956

[ref40] Jost J. T. Glaser J. Kruglanski A. W. Sulloway F. J. (2003). Political conservatism as motivated social cognition. Psychol. Bull. 129, 339–375. doi: 10.1037/0033-2909.129.3.339 12784934

[ref41] Kable J. W. (2011). The cognitive neuroscience toolkit for the Neuroeconomist: a functional overview. J. Neurosci. Psychol. Econ. 4, 63–84. doi: 10.1037/a0023555, PMID: 21796272 PMC3142554

[ref42] Kahneman D. (2003). Maps of bounded rationality: psychology for behavioral economics. Am. Econ. Rev. 93, 1449–1475. doi: 10.1257/000282803322655392

[ref43] Kahneman D. Knetsch J. L. Thaler R. H. (1991). Anomalies the endowment effect, loss aversion, and status quo bias. J. Econ. Perspect. 5, 193–206. doi: 10.1257/jep.5.1.193

[ref44] Kruglanski A. W. Gigerenzer G. (2011). Intuitive and deliberate judgments are based on common principles. Psychol. Rev. 118, 97–109. doi: 10.1037/a0020762, PMID: 21244188

[ref45] Kruglanski A. W. Szumowska E. Kopetz C. H. Vallerand R. J. Pierro A. (2021). On the psychology of extremism: how motivational imbalance breeds intemperance. Psychol. Rev. 128, 264–289. doi: 10.1037/REV0000260 32915010

[ref46] Kung F. Y. H. Scholer A. A. (2021). Moving beyond two goals: an integrative review and framework for the study of multiple goals. Personal. Soc. Psychol. Rev. 25, 130–158. doi: 10.1177/1088868320985810 33538215

[ref47] Kurdoglu R. S. Ateş N. Y. (2022). Arguing to defeat: eristic argumentation and irrationality in resolving moral concerns. J. Bus. Ethics 175, 519–535. doi: 10.1007/s10551-020-04659-2

[ref48] Kurdoglu R. S. Ates N. Y. Lerner D. A. (2023). Decision-making under extreme uncertainty: eristic rather than heuristic. Int. J. Entrepreneurial Behav. Res. doi: 10.1108/IJEBR-07-2022-0587 (Epub ahead of print)

[ref49] Kurdoglu R. S. Lerner D. Ates N. Y. (2022). Unsticking the rationality stalemate: motivated reasoning, reality, and irrationality. J. Bus. Ventur. Insights 18:e00336. doi: 10.1016/J.JBVI.2022.E00336

[ref50] Lex M. Gielnik M. M. Spitzmuller M. Jacob G. H. Frese M. (2022). How passion in entrepreneurship develops over time: a self-regulation perspective. Entrep. Theory Pract. 46, 985–1018. doi: 10.1177/1042258720929894

[ref51] Lieder F. Griffiths T. L. (2020). Resource-rational analysis: understanding human cognition as the optimal use of limited computational resources. Behav. Brain Sci. 43:e1. doi: 10.1017/S0140525X1900061X, PMID: 30714890

[ref52] Loewenstein G. Rick S. Cohen J. D. (2008). Neuroeconomics. Annu. Rev. Psychol. 59, 647–672. doi: 10.1146/annurev.psych.59.103006.093710 17883335

[ref53] Martela F. (2015). Fallible inquiry with ethical ends-in-view: a pragmatist philosophy of science for organizational research. Organ. Stud. 36, 537–563. doi: 10.1177/0170840614559257

[ref54] Mercier H. (2013). The function of reasoning: argumentative and pragmatic alternatives. Think. Reason. 19, 488–494. doi: 10.1080/13546783.2013.819036

[ref55] Mercier H. Sperber D. (2011). Why do humans reason? Arguments for an argumentative theory. Behav. Brain Sci. 34, 57–74. doi: 10.1017/S0140525X10000968 21447233

[ref56] Mises L. Von (1988). Human action: A treatise on economics. Auburn, ALABAMA: Ludwig von Mises Institute.

[ref57] Mishra S. (2014). Decision-making under risk: integrating perspectives from biology, economics, and psychology. Personal. Soc. Psychol. Rev. 18, 280–307. doi: 10.1177/1088868314530517 24769798

[ref58] Morisseau T. Branch T. Y. Origgi G. (2021). Stakes of knowing the truth: a motivational perspective on the popularity of a controversial scientific theory. Front. Psychol. 12:708751. doi: 10.3389/FPSYG.2021.708751/BIBTEX 34603134 PMC8485728

[ref59] Mueller B. A. Wolfe M. T. Syed I. (2017). Passion and grit: an exploration of the pathways leading to venture success. J. Bus. Ventur. 32, 260–279. doi: 10.1016/j.jbusvent.2017.02.001

[ref60] Navarrete G. Santamaría C. (2011). Ecological rationality and evolution: the mind really works that way? Front. Psychol. 2:251. doi: 10.3389/FPSYG.2011.00251/BIBTEX 21994499 PMC3183440

[ref61] Newell B. R. Weston N. J. Shanks D. R. (2003). Empirical tests of a fast-and-frugal heuristic: not everyone ‘takes-the-best’. Organ. Behav. Hum. Decis. Process. 91, 82–96. doi: 10.1016/S0749-5978(02)00525-3

[ref62] Nicolaou N. Phan P. H. Stephan U. (2021). The biological perspective in entrepreneurship research. Entrep. Theory Pract. 45, 3–17. doi: 10.1177/1042258720967314

[ref63] Packard M. D. Clark B. B. Kleinc P. G. (2017). Uncertainty types and transitions in the entrepreneurial process. Organ. Sci. 28, 840–856. doi: 10.1287/orsc.2017.1143

[ref64] Peirce C. S. (1997). Pragmatism as a principle and method of right thinking: The 1903 Harvard lectures on pragmatism. Albany, NY: State University of New York Press.

[ref65] Perelman C. (1979). The new rhetoric and the humanities: Essays on rhetoric and its applications. Dordrecht: D. Reidel Publishing Company.

[ref66] Perelman C. (1982). The realm of rhetoric. London: University of Notre Dame Press.

[ref67] Perelman C Olbrechts-Tyteca L. (1969). The new rhetoric: A treatise on argumentation. E-book ver. London: University of Notre Dame Press.

[ref68] Perry-Smith J. E. Mannucci P. V. (2017). From creativity to innovation: the social network drivers of the four phases of the idea journey. Acad. Manag. Rev. 42, 53–79. doi: 10.5465/amr.2014.0462

[ref69] Risen J. L. (2016). Believing what we do not believe: acquiescence to superstitious beliefs and other powerful intuitions. Psychol. Rev. 123, 182–207. doi: 10.1037/rev0000017 26479707

[ref70] Sanfey A. G. Loewenstein G. McClure S. M. Cohen J. D. (2006). Neuroeconomics: cross-currents in research on decision-making. Trends Cogn. Sci. 10, 108–116. doi: 10.1016/j.tics.2006.01.009, PMID: 16469524

[ref71] Serra D. (2021). Decision-making: from neuroscience to neuroeconomics—an overview. Theor. Decis. 91, 1–80. doi: 10.1007/S11238-021-09830-3/FIGURES/6

[ref72] Seybert N. Bloomfield R. (2009). Contagion of wishful thinking in markets. Manag. Sci. 55, 738–751. doi: 10.1287/mnsc.l080.0973

[ref73] Shafir E. Tversky A. (1992). Thinking through uncertainty: nonconsequential reasoning and choice. Cogn. Psychol. 24, 449–474. doi: 10.1016/0010-0285(92)90015-T, PMID: 1473331

[ref74] Shah A. K. Oppenheimer D. M. (2008). Heuristics made easy: an effort-reduction framework. Psychol. Bull. 134, 207–222. doi: 10.1037/0033-2909.134.2.207, PMID: 18298269

[ref75] Sharma L. Markon K. E. Clark L. A. (2014). Toward a theory of distinct types of ‘impulsive’ behaviors: a meta-analysis of self-report and behavioral measures. Psychol. Bull. 140, 374–408. doi: 10.1037/a0034418 24099400

[ref76] Simon H. A. (1978). Rationality as process and as product of thought. Am. Econ. Rev. 1978, 1–16.

[ref77] Simon H. A. (1983). Reason in human affairs. Stanford, CA: Stanford University Press.

[ref78] Simon H. A. (1990). Invariants of human behavior. Annu. Rev. Psychol. 41, 1–20. doi: 10.1146/ANNUREV.PS.41.020190.000245 18331187

[ref79] Tetlock P. E. (2002). Social functionalist frameworks for judgment and choice: intuitive politicians, theologians, and prosecutors. Psychol. Rev. 109, 451–471. doi: 10.1037/0033-295X.109.3.451, PMID: 12088240

[ref80] Tsang E. W. K. (2011). Superstition and decision-making: contradiction or complement? Acad. Manag. Exec. 18, 92–104. doi: 10.5465/ame.2004.15268696

[ref81] Vail K. E. III Juhl J. Arndt J. Vess M. Routledge C. Rutjens B. T. (2012). When death is good for life: considering the positive trajectories of terror management. Personal. Soc. Psychol. Rev. 16, 303–329. doi: 10.1177/1088868312440046 22490977

[ref82] Vallerand R. J. Blanchard C. Mageau G. A. Koestner R. Ratelle C. Léonard M.. (2003). Les passions de 1′Âme: on obsessive and harmonious passion. J. Pers. Soc. Psychol. 85, 756–767. doi: 10.1037/0022-3514.85.4.756, PMID: 14561128

[ref83] Volk S. Köhler T. (2012). Brains and games: applying neuroeconomics to organizational research. Organ. Res. Methods 15, 522–552. doi: 10.1177/1094428112449656

[ref84] Walco D. K. Risen J. L. (2017). The empirical case for acquiescing to intuition. Psychol. Sci. 28, 1807–1820. doi: 10.1177/0956797617723377, PMID: 29040055

[ref85] Wolf S. (2010). A system of argumentation forms in Aristotle. Argumentation 24, 19–40. doi: 10.1007/s10503-009-9127-1

